# Supplementary material for: Comparative multiomics analysis of cell physiological state after culture in a basket bioreactor
Source: Sci Rep. 2022 Nov 23;12:20161. doi: 10.1038/s41598-022-24687-4 (PMC9686226; doi:10.1038/s41598-022-24687-4)
Supplement: Supplementary file 1 — Supplementary Information 1. [file 41598_2022_24687_MOESM1_ESM.zip › raw data/Transcriptomic raw data/6.Enrichment/2.KEGG/TE1vsTC1/DOWN/TE1vsTC1.down_KEGGenrich.html]

Pathway Enrichment


# The most enriched pathway terms

Statistic method: hypergeometric test

FDR correction method: Benjamini and Hochberg

| Term | Description | Sample number | Background number | P-value | Corrected P-value | Gene\_id | Gene\_name | KEGG\_ID |
| --- | --- | --- | --- | --- | --- | --- | --- | --- |
| csab04621 | NOD-like receptor signaling pathway | 10/88 | 131/5900 | 2.14245748231631e-05 | 0.00471340646109589 | 103224057 103241278 103224476 103239154 103230198 103235789 103224480 103215179 103242710 103239156 | LOC103224057 IL1B LOC103224476 OAS1 IKBKE CXCL1 LOC103224480 CARD6 CCL2 OAS2 | csab:103224057 csab:103241278 csab:103224476 csab:103239154 csab:103230198 csab:103235798 csab:103224480 csab:103215179 csab:103242710 csab:103239156 |
| csab04060 | Cytokine-cytokine receptor interaction | 10/88 | 170/5900 | 0.000195994920761569 | 0.0215594412837726 | 103241278 103237005 103219268 103235789 103225587 103242551 103242198 103225819 103223920 103242710 | IL1B IL7 CNTFR CXCL1 TNFRSF1B TNFRSF6B CXCL16 TNFRSF18 IL6R CCL2 | csab:103241278 csab:103237005 csab:103219268 csab:103235798 csab:103225587 csab:103242551 csab:103242198 csab:103225819 csab:103223920 csab:103242710 |
| csab05171 | Coronavirus disease - COVID-19 | 9/88 | 163/5900 | 0.000662076882493122 | 0.0485523047161623 | 103241278 103221732 103239154 103230198 103224900 103233571 103223920 103242710 103239156 | IL1B LOC103221732 OAS1 IKBKE PIK3R3 CFD IL6R CCL2 OAS2 | csab:103241278 csab:103221732 csab:103239154 csab:103230198 csab:103224900 csab:103233571 csab:103223920 csab:103242710 csab:103239156 |
| csab04610 | Complement and coagulation cascades | 5/88 | 55/5900 | 0.00127392656917174 | 0.0700659613044457 | 103229609 103231401 103215685 103221732 103233571 | SERPINA1 ITGAX PLAT LOC103221732 CFD | csab:103229609 csab:103231401 csab:103215685 csab:103221732 csab:103233571 |
| csab05164 | Influenza A | 7/88 | 116/5900 | 0.00161613062853756 | 0.0711097476556526 | 103241278 103239154 103230198 103224900 103232396 103242710 103239156 | IL1B OAS1 IKBKE PIK3R3 NXF3 CCL2 OAS2 | csab:103241278 csab:103239154 csab:103230198 csab:103224900 csab:103232396 csab:103242710 csab:103239156 |
| csab05417 | Lipid and atherosclerosis | 8/88 | 160/5900 | 0.00250487492697533 | 0.0918454139890953 | 103241278 103240818 103230198 103235789 103223257 103224900 103223677 103242710 | IL1B SOD2 IKBKE CXCL1 NCF4 PIK3R3 HSPA6 CCL2 | csab:103241278 csab:103240818 csab:103230198 csab:103235798 csab:103223257 csab:103224900 csab:103223677 csab:103242710 |
| csab04668 | TNF signaling pathway | 6/88 | 98/5900 | 0.00325823758153047 | 0.100649222832222 | 103241278 103235789 103225587 103218917 103224900 103242710 | IL1B CXCL1 TNFRSF1B TRAF1 PIK3R3 CCL2 | csab:103241278 csab:103235798 csab:103225587 csab:103218917 csab:103224900 csab:103242710 |
| csab00590 | Arachidonic acid metabolism | 4/88 | 43/5900 | 0.00365997173935354 | 0.100649222832222 | 103235528 103245108 103218549 103220573 | PLA2G4C LTC4S CBR3 PLB1 | csab:103235528 csab:103245108 csab:103218549 csab:103220573 |
| csab05162 | Measles | 6/88 | 110/5900 | 0.00575236995929362 | 0.126226097105248 | 103241278 103239154 103230198 103224900 103223677 103239156 | IL1B OAS1 IKBKE PIK3R3 HSPA6 OAS2 | csab:103241278 csab:103239154 csab:103230198 csab:103224900 csab:103223677 csab:103239156 |
| csab05418 | Fluid shear stress and atherosclerosis | 6/88 | 111/5900 | 0.00600964672087665 | 0.126226097105248 | 103241278 103215685 103220724 103218213 103224900 103242710 | IL1B PLAT SDC1 GPC1 PIK3R3 CCL2 | csab:103241278 csab:103215685 csab:103220724 csab:103218213 csab:103224900 csab:103242710 |
| csab04061 | Viral protein interaction with cytokine and cytokine receptor | 4/88 | 50/5900 | 0.00631130485526241 | 0.126226097105248 | 103235789 103225587 103223920 103242710 | CXCL1 TNFRSF1B IL6R CCL2 | csab:103235798 csab:103225587 csab:103223920 csab:103242710 |
| csab05144 | Malaria | 3/88 | 35/5900 | 0.0148753451244315 | 0.271712606300945 | 103241278 103220724 103242710 | IL1B SDC1 CCL2 | csab:103241278 csab:103220724 csab:103242710 |
| csab00565 | Ether lipid metabolism | 3/88 | 36/5900 | 0.0160557449177831 | 0.271712606300945 | 103235528 103224742 103220573 | PLA2G4C PLPP3 PLB1 | csab:103235528 csab:103224742 csab:103220573 |
| csab04657 | IL-17 signaling pathway | 4/88 | 69/5900 | 0.0191425502228207 | 0.298614847851003 | 103241278 103230198 103235789 103242710 | IL1B IKBKE CXCL1 CCL2 | csab:103241278 csab:103230198 csab:103235798 csab:103242710 |
| csab00592 | alpha-Linolenic acid metabolism | 2/88 | 15/5900 | 0.0203601032625684 | 0.298614847851003 | 103235528 103220573 | PLA2G4C PLB1 | csab:103235528 csab:103220573 |
| csab00591 | Linoleic acid metabolism | 2/88 | 16/5900 | 0.0230457890248556 | 0.316879599091765 | 103235528 103220573 | PLA2G4C PLB1 | csab:103235528 csab:103220573 |
| csab04630 | JAK-STAT signaling pathway | 5/88 | 111/5900 | 0.0245341876503301 | 0.317501251945448 | 103237005 103218491 103219268 103224900 103223920 | IL7 PTPN6 CNTFR PIK3R3 IL6R | csab:103237005 csab:103218491 csab:103219268 csab:103224900 csab:103223920 |
| csab05134 | Legionellosis | 3/88 | 44/5900 | 0.0273567674490609 | 0.334360491044078 | 103241278 103235789 103223677 | IL1B CXCL1 HSPA6 | csab:103241278 csab:103235798 csab:103223677 |
| csab00564 | Glycerophospholipid metabolism | 4/88 | 80/5900 | 0.0309799788075936 | 0.358715544087926 | 103235528 103228224 103224742 103220573 | PLA2G4C PLA1A PLPP3 PLB1 | csab:103235528 csab:103228224 csab:103224742 csab:103220573 |
| csab04066 | HIF-1 signaling pathway | 4/88 | 83/5900 | 0.0348154783364708 | 0.367870262779581 | 103242625 103218486 103224900 103223920 | ALDOC ENO2 PIK3R3 IL6R | csab:103242625 csab:103218486 csab:103224900 csab:103223920 |
| csab00770 | Pantothenate and CoA biosynthesis | 2/88 | 20/5900 | 0.0351148887198691 | 0.367870262779581 | 103240354 103240351 | LOC103240354 VNN2 | csab:103240354 csab:103240351 |
| csab04940 | Type I diabetes mellitus | 2/88 | 21/5900 | 0.0384415663259925 | 0.380082561364586 | 103241278 103217907 | IL1B PTPRN | csab:103241278 csab:103217907 |
| csab05205 | Proteoglycans in cancer | 6/88 | 169/5900 | 0.0397359041426613 | 0.380082561364586 | 103223483 103218491 103220724 103224207 103218213 103224900 | WNT7B PTPN6 SDC1 WNT2B GPC1 PIK3R3 | csab:103223483 csab:103218491 csab:103220724 csab:103224207 csab:103218213 csab:103224900 |
| csab04213 | Longevity regulating pathway - multiple species | 3/88 | 53/5900 | 0.044004257476898 | 0.403372360204898 | 103240818 103224900 103223677 | SOD2 PIK3R3 HSPA6 | csab:103240818 csab:103224900 csab:103223677 |
| csab05140 | Leishmaniasis | 3/88 | 54/5900 | 0.04610538091073 | 0.405727352014424 | 103241278 103218491 103223257 | IL1B PTPN6 NCF4 | csab:103241278 csab:103218491 csab:103223257 |
| csab04640 | Hematopoietic cell lineage | 3/88 | 56/5900 | 0.050454493549737 | 0.426922637728544 | 103241278 103237005 103223920 | IL1B IL7 IL6R | csab:103241278 csab:103237005 csab:103223920 |
| csab04014 | Ras signaling pathway | 6/88 | 184/5900 | 0.0559965953450569 | 0.449943151292473 | 103230197 103246479 103235528 103228224 103224900 103223351 | RASSF5 FGFR3 PLA2G4C PLA1A PIK3R3 RIN1 | csab:103230197 csab:103246479 csab:103235528 csab:103228224 csab:103224900 csab:103223351 |
| csab04721 | Synaptic vesicle cycle | 3/88 | 60/5900 | 0.0597283704509865 | 0.449943151292473 | 103246629 103246502 103239806 | STX1A SNAP25 DNM1 | csab:103246629 csab:103246502 csab:103239806 |
| csab04960 | Aldosterone-regulated sodium reabsorption | 2/88 | 27/5900 | 0.0606816283870028 | 0.449943151292473 | 103224900 103229709 | PIK3R3 SCNN1B | csab:103224900 csab:103229709 |
| csab05323 | Rheumatoid arthritis | 3/88 | 61/5900 | 0.0621640829771992 | 0.449943151292473 | 103241278 103235789 103242710 | IL1B CXCL1 CCL2 | csab:103241278 csab:103235798 csab:103242710 |
| csab04936 | Alcoholic liver disease | 4/88 | 101/5900 | 0.063401080409394 | 0.449943151292473 | 103241278 103221732 103230198 103235789 | IL1B LOC103221732 IKBKE CXCL1 | csab:103241278 csab:103221732 csab:103230198 csab:103235798 |
| csab04911 | Insulin secretion | 3/88 | 64/5900 | 0.0697445581919509 | 0.479493837569662 | 103226636 103246629 103246502 | PCLO STX1A SNAP25 | csab:103226636 csab:103246629 csab:103246502 |
| csab01521 | EGFR tyrosine kinase inhibitor resistance | 3/88 | 69/5900 | 0.0832568031483912 | 0.546650078303232 | 103246479 103224900 103223920 | FGFR3 PIK3R3 IL6R | csab:103246479 csab:103224900 csab:103223920 |
| csab05169 | Epstein-Barr virus infection | 5/88 | 157/5900 | 0.0844822848286814 | 0.546650078303232 | 103239154 103230198 103224900 103221664 103239156 | OAS1 IKBKE PIK3R3 LOC103221664 OAS2 | csab:103239154 csab:103230198 csab:103224900 csab:103221664 csab:103239156 |
| csab04550 | Signaling pathways regulating pluripotency of stem cells | 4/88 | 116/5900 | 0.094335112088572 | 0.553667119477461 | 103223483 103246479 103224207 103224900 | WNT7B FGFR3 WNT2B PIK3R3 | csab:103223483 csab:103246479 csab:103224207 csab:103224900 |
| csab00830 | Retinol metabolism | 2/88 | 35/5900 | 0.0953758659152141 | 0.553667119477461 | 103236970 103225584 | RDH10 DHRS3 | csab:103236970 csab:103225584 |
| csab04620 | Toll-like receptor signaling pathway | 3/88 | 75/5900 | 0.100828990205663 | 0.553667119477461 | 103241278 103230198 103224900 | IL1B IKBKE PIK3R3 | csab:103241278 csab:103230198 csab:103224900 |
| csab05146 | Amoebiasis | 3/88 | 75/5900 | 0.100828990205663 | 0.553667119477461 | 103241278 103235789 103224900 | IL1B CXCL1 PIK3R3 | csab:103241278 csab:103235798 csab:103224900 |
| csab05200 | Pathways in cancer | 10/88 | 428/5900 | 0.103110138326254 | 0.553667119477461 | 103220342 103237005 103230197 103223483 103246479 103224207 103218917 103224900 103239621 103223920 | EPAS1 IL7 RASSF5 WNT7B FGFR3 WNT2B TRAF1 PIK3R3 NOTCH1 IL6R | csab:103220342 csab:103237005 csab:103230197 csab:103223483 csab:103246479 csab:103224207 csab:103218917 csab:103224900 csab:103239621 csab:103223920 |
| csab04666 | Fc gamma R-mediated phagocytosis | 3/88 | 77/5900 | 0.106993620821797 | 0.553667119477461 | 103235528 103224742 103224900 | PLA2G4C PLPP3 PIK3R3 | csab:103235528 csab:103224742 csab:103224900 |
| csab04750 | Inflammatory mediator regulation of TRP channels | 3/88 | 78/5900 | 0.110130594476337 | 0.553667119477461 | 103241278 103235528 103224900 | IL1B PLA2G4C PIK3R3 | csab:103241278 csab:103235528 csab:103224900 |
| csab04064 | NF-kappa B signaling pathway | 3/88 | 79/5900 | 0.11330311565909 | 0.553667119477461 | 103241278 103235789 103218917 | IL1B CXCL1 TRAF1 | csab:103241278 csab:103235798 csab:103218917 |
| csab04623 | Cytosolic DNA-sensing pathway | 2/88 | 39/5900 | 0.114405610827303 | 0.553667119477461 | 103241278 103230198 | IL1B IKBKE | csab:103241278 csab:103230198 |
| csab05163 | Human cytomegalovirus infection | 5/88 | 173/5900 | 0.115309617619517 | 0.553667119477461 | 103241278 103224900 103221664 103223920 103242710 | IL1B PIK3R3 LOC103221664 IL6R CCL2 | csab:103241278 csab:103224900 csab:103221664 csab:103223920 csab:103242710 |
| csab05160 | Hepatitis C | 4/88 | 125/5900 | 0.115766761345287 | 0.553667119477461 | 103239154 103230198 103224900 103239156 | OAS1 IKBKE PIK3R3 OAS2 | csab:103239154 csab:103230198 csab:103224900 csab:103239156 |
| csab05224 | Breast cancer | 4/88 | 125/5900 | 0.115766761345287 | 0.553667119477461 | 103223483 103224207 103224900 103239621 | WNT7B WNT2B PIK3R3 NOTCH1 | csab:103223483 csab:103224207 csab:103224900 csab:103239621 |
| csab04072 | Phospholipase D signaling pathway | 4/88 | 128/5900 | 0.12335234503609 | 0.566419265429539 | 103235528 103224742 103224900 103239806 | PLA2G4C PLPP3 PIK3R3 DNM1 | csab:103235528 csab:103224742 csab:103224900 csab:103239806 |
| csab04625 | C-type lectin receptor signaling pathway | 3/88 | 83/5900 | 0.126335149043619 | 0.566419265429539 | 103241278 103230198 103224900 | IL1B IKBKE PIK3R3 | csab:103241278 csab:103230198 csab:103224900 |
| csab05231 | Choline metabolism in cancer | 3/88 | 85/5900 | 0.133046783446898 | 0.566419265429539 | 103235528 103224742 103224900 | PLA2G4C PLPP3 PIK3R3 | csab:103235528 csab:103224742 csab:103224900 |
| csab04150 | mTOR signaling pathway | 4/88 | 132/5900 | 0.133790169925596 | 0.566419265429539 | 103223483 103224207 103246611 103224900 | WNT7B WNT2B CASTOR2 PIK3R3 | csab:103223483 csab:103224207 csab:103246611 csab:103224900 |
| csab04612 | Antigen processing and presentation | 2/88 | 43/5900 | 0.134309495801336 | 0.566419265429539 | 103223677 103221664 | HSPA6 LOC103221664 | csab:103223677 csab:103221664 |
| csab05142 | Chagas disease | 3/88 | 86/5900 | 0.136449102076159 | 0.566419265429539 | 103241278 103224900 103242710 | IL1B PIK3R3 CCL2 | csab:103241278 csab:103224900 csab:103242710 |
| csab04062 | Chemokine signaling pathway | 4/88 | 133/5900 | 0.136455550308025 | 0.566419265429539 | 103235789 103242198 103224900 103242710 | CXCL1 CXCL16 PIK3R3 CCL2 | csab:103235798 csab:103242198 csab:103224900 csab:103242710 |
| csab00600 | Sphingolipid metabolism | 2/88 | 44/5900 | 0.13940248286573 | 0.567936041304826 | 103224742 103233234 | PLPP3 SMPD3 | csab:103224742 csab:103233234 |
| csab00980 | Metabolism of xenobiotics by cytochrome P450 | 2/88 | 45/5900 | 0.144537377531143 | 0.578149510124573 | 103230154 103218549 | HSD11B1 CBR3 | csab:103230154 csab:103218549 |
| csab00430 | Taurine and hypotaurine metabolism | 1/88 | 11/5900 | 0.152483500877259 | 0.591158208009048 | 103230592 | FMO4 | csab:103230592 |
| csab05150 | Staphylococcus aureus infection | 2/88 | 47/5900 | 0.154923578824058 | 0.591158208009048 | 103221732 103233571 | LOC103221732 CFD | csab:103221732 csab:103233571 |
| csab05168 | Herpes simplex virus 1 infection | 9/88 | 410/5900 | 0.155907586334101 | 0.591158208009048 | 103241278 103239154 103230198 103224900 103232396 103221664 103219115 103242710 103239156 | IL1B OAS1 IKBKE PIK3R3 NXF3 LOC103221664 ZNF189 CCL2 OAS2 | csab:103241278 csab:103239154 csab:103230198 csab:103224900 csab:103232396 csab:103221664 csab:103219115 csab:103242710 csab:103239156 |
| csab04310 | Wnt signaling pathway | 4/88 | 141/5900 | 0.158537883056972 | 0.591158208009048 | 103223483 103215672 103224207 103237471 | WNT7B SFRP1 WNT2B CCN4 | csab:103223483 csab:103215672 csab:103224207 csab:103237471 |
| csab04370 | VEGF signaling pathway | 2/88 | 49/5900 | 0.165450061651346 | 0.606650226054935 | 103235528 103224900 | PLA2G4C PIK3R3 | csab:103235528 csab:103224900 |
| csab04933 | AGE-RAGE signaling pathway in diabetic complications | 3/88 | 95/5900 | 0.168340850010563 | 0.607130934464326 | 103241278 103224900 103242710 | IL1B PIK3R3 CCL2 | csab:103241278 csab:103224900 csab:103242710 |
| csab04270 | Vascular smooth muscle contraction | 3/88 | 99/5900 | 0.183165843551997 | 0.622049071775388 | 103235528 103240389 103223064 | PLA2G4C ADM ADORA2A | csab:103235528 csab:103240389 csab:103223064 |
| csab00010 | Glycolysis / Gluconeogenesis | 2/88 | 53/5900 | 0.186856219309638 | 0.622049071775388 | 103242625 103218486 | ALDOC ENO2 | csab:103242625 csab:103218486 |
| csab05133 | Pertussis | 2/88 | 53/5900 | 0.186856219309638 | 0.622049071775388 | 103241278 103221732 | IL1B LOC103221732 | csab:103241278 csab:103221732 |
| csab04977 | Vitamin digestion and absorption | 1/88 | 14/5900 | 0.189918455718387 | 0.622049071775388 | 103220573 | PLB1 | csab:103220573 |
| csab04380 | Osteoclast differentiation | 3/88 | 101/5900 | 0.190708126874442 | 0.622049071775388 | 103241278 103223257 103224900 | IL1B NCF4 PIK3R3 | csab:103241278 csab:103223257 csab:103224900 |
| csab04670 | Leukocyte transendothelial migration | 3/88 | 101/5900 | 0.190708126874442 | 0.622049071775388 | 103230197 103223257 103224900 | RASSF5 NCF4 PIK3R3 | csab:103230197 csab:103223257 csab:103224900 |
| csab04664 | Fc epsilon RI signaling pathway | 2/88 | 54/5900 | 0.192269713094211 | 0.622049071775388 | 103235528 103224900 | PLA2G4C PIK3R3 | csab:103235528 csab:103224900 |
| csab05217 | Basal cell carcinoma | 2/88 | 56/5900 | 0.203157901375916 | 0.628745043315923 | 103223483 103224207 | WNT7B WNT2B | csab:103223483 csab:103224207 |
| csab04919 | Thyroid hormone signaling pathway | 3/88 | 105/5900 | 0.206025568286545 | 0.628745043315923 | 103232196 103224900 103239621 | SLC16A2 PIK3R3 NOTCH1 | csab:103232196 csab:103224900 csab:103239621 |
| csab05020 | Prion disease | 5/88 | 211/5900 | 0.206068497126802 | 0.628745043315923 | 103241278 103223257 103224900 103239621 103223677 | IL1B NCF4 PIK3R3 NOTCH1 HSPA6 | csab:103241278 csab:103223257 csab:103224900 csab:103239621 csab:103223677 |
| csab01230 | Biosynthesis of amino acids | 2/88 | 57/5900 | 0.208629037100284 | 0.628745043315923 | 103242625 103218486 | ALDOC ENO2 | csab:103242625 csab:103218486 |
| csab04662 | B cell receptor signaling pathway | 2/88 | 57/5900 | 0.208629037100284 | 0.628745043315923 | 103218491 103224900 | PTPN6 PIK3R3 | csab:103218491 csab:103224900 |
| csab04210 | Apoptosis | 3/88 | 107/5900 | 0.213790225389252 | 0.635592561968047 | 103218917 103245241 103224900 | TRAF1 CTSH PIK3R3 | csab:103218917 csab:103245241 csab:103224900 |
| csab05230 | Central carbon metabolism in cancer | 2/88 | 60/5900 | 0.225130244324038 | 0.660382050017177 | 103246479 103224900 | FGFR3 PIK3R3 | csab:103246479 csab:103224900 |
| csab05211 | Renal cell carcinoma | 2/88 | 61/5900 | 0.230654520979862 | 0.667056124212146 | 103220342 103224900 | EPAS1 PIK3R3 | csab:103220342 csab:103224900 |
| csab05135 | Yersinia infection | 3/88 | 112/5900 | 0.233469643474251 | 0.667056124212146 | 103241278 103224900 103242710 | IL1B PIK3R3 CCL2 | csab:103241278 csab:103224900 csab:103242710 |
| csab05332 | Graft-versus-host disease | 1/88 | 18/5900 | 0.237299854824618 | 0.669307282838667 | 103241278 | IL1B | csab:103241278 |
| csab05223 | Non-small cell lung cancer | 2/88 | 63/5900 | 0.241729638136291 | 0.673171144177013 | 103230197 103224900 | RASSF5 PIK3R3 | csab:103230197 csab:103224900 |
| csab05320 | Autoimmune thyroid disease | 1/88 | 19/5900 | 0.248710533637171 | 0.682287191041941 | 103237469 | TG | csab:103237469 |
| csab04068 | FoxO signaling pathway | 3/88 | 117/5900 | 0.253469347869768 | 0.682287191041941 | 103240818 103245773 103224900 | SOD2 SGK2 PIK3R3 | csab:103240818 csab:103245773 csab:103224900 |
| csab04015 | Rap1 signaling pathway | 4/88 | 172/5900 | 0.254307043933814 | 0.682287191041941 | 103230197 103246479 103223064 103224900 | RASSF5 FGFR3 ADORA2A PIK3R3 | csab:103230197 csab:103246479 csab:103223064 csab:103224900 |
| csab05100 | Bacterial invasion of epithelial cells | 2/88 | 66/5900 | 0.258387018001193 | 0.683154072720594 | 103224900 103239806 | PIK3R3 DNM1 | csab:103224900 csab:103239806 |
| csab04142 | Lysosome | 3/88 | 119/5900 | 0.261542008497981 | 0.683154072720594 | 103242313 103245241 103243148 | CD68 CTSH ARSG | csab:103242313 csab:103245241 csab:103243148 |
| csab04650 | Natural killer cell mediated cytotoxicity | 2/88 | 67/5900 | 0.263945891732957 | 0.683154072720594 | 103218491 103224900 | PTPN6 PIK3R3 | csab:103218491 csab:103224900 |
| csab00650 | Butanoate metabolism | 1/88 | 21/5900 | 0.271027951853423 | 0.685358039169575 | 103227954 | ABAT | csab:103227954 |
| csab05143 | African trypanosomiasis | 1/88 | 21/5900 | 0.271027951853423 | 0.685358039169575 | 103241278 | IL1B | csab:103241278 |
| csab00515 | Mannose type O-glycan biosynthesis | 1/88 | 22/5900 | 0.281939593329337 | 0.702545330942485 | 103242981 | MGAT5B | csab:103242981 |
| csab04810 | Regulation of actin cytoskeleton | 4/88 | 181/5900 | 0.284211520244914 | 0.702545330942485 | 103231401 103246479 103224900 103223856 | ITGAX FGFR3 PIK3R3 IQGAP3 | csab:103231401 csab:103246479 csab:103224900 csab:103223856 |
| csab04975 | Fat digestion and absorption | 1/88 | 23/5900 | 0.292689732115832 | 0.706379023733005 | 103224742 | PLPP3 | csab:103224742 |
| csab05226 | Gastric cancer | 3/88 | 127/5900 | 0.294133658674063 | 0.706379023733005 | 103223483 103224207 103224900 | WNT7B WNT2B PIK3R3 | csab:103223483 csab:103224207 csab:103224900 |
| csab05235 | PD-L1 expression and PD-1 checkpoint pathway in cancer | 2/88 | 74/5900 | 0.302819051026284 | 0.706379023733005 | 103218491 103224900 | PTPN6 PIK3R3 | csab:103218491 csab:103224900 |
| csab00030 | Pentose phosphate pathway | 1/88 | 24/5900 | 0.303280731532849 | 0.706379023733005 | 103242625 | ALDOC | csab:103242625 |
| csab04010 | MAPK signaling pathway | 5/88 | 247/5900 | 0.307824301096488 | 0.706379023733005 | 103241278 103246479 103235528 103225290 103223677 | IL1B FGFR3 PLA2G4C MAP3K6 HSPA6 | csab:103241278 csab:103246479 csab:103235528 csab:103225290 csab:103223677 |
| csab01523 | Antifolate resistance | 1/88 | 25/5900 | 0.313714920713432 | 0.706379023733005 | 103241278 | IL1B | csab:103241278 |
| csab04211 | Longevity regulating pathway | 2/88 | 76/5900 | 0.313878975590573 | 0.706379023733005 | 103240818 103224900 | SOD2 PIK3R3 | csab:103240818 csab:103224900 |
| csab04916 | Melanogenesis | 2/88 | 76/5900 | 0.313878975590573 | 0.706379023733005 | 103223483 103224207 | WNT7B WNT2B | csab:103223483 csab:103224207 |
| csab04932 | Non-alcoholic fatty liver disease | 3/88 | 132/5900 | 0.314659746935611 | 0.706379023733005 | 103241278 103224900 103223920 | IL1B PIK3R3 IL6R | csab:103241278 csab:103224900 csab:103223920 |
| csab04660 | T cell receptor signaling pathway | 2/88 | 80/5900 | 0.335883319115323 | 0.734969625511023 | 103218491 103224900 | PTPN6 PIK3R3 | csab:103218491 csab:103224900 |
| csab04659 | Th17 cell differentiation | 2/88 | 81/5900 | 0.341354995499763 | 0.734969625511023 | 103241278 103223920 | IL1B IL6R | csab:103241278 csab:103223920 |
| csab00250 | Alanine, aspartate and glutamate metabolism | 1/88 | 28/5900 | 0.344099415580161 | 0.734969625511023 | 103227954 | ABAT | csab:103227954 |
| csab00410 | beta-Alanine metabolism | 1/88 | 28/5900 | 0.344099415580161 | 0.734969625511023 | 103227954 | ABAT | csab:103227954 |
| csab00640 | Propanoate metabolism | 1/88 | 28/5900 | 0.344099415580161 | 0.734969625511023 | 103227954 | ABAT | csab:103227954 |
| csab05215 | Prostate cancer | 2/88 | 85/5900 | 0.363098909367272 | 0.747748964624573 | 103215685 103224900 | PLAT PIK3R3 | csab:103215685 csab:103224900 |
| csab05206 | MicroRNAs in cancer | 3/88 | 145/5900 | 0.368086039693897 | 0.747748964624573 | 103246479 103224900 103239621 | FGFR3 PIK3R3 NOTCH1 | csab:103246479 csab:103224900 csab:103239621 |
| csab05225 | Hepatocellular carcinoma | 3/88 | 146/5900 | 0.372179209025432 | 0.747748964624573 | 103223483 103224207 103224900 | WNT7B WNT2B PIK3R3 | csab:103223483 csab:103224207 csab:103224900 |
| csab04672 | Intestinal immune network for IgA production | 1/88 | 31/5900 | 0.373153286472273 | 0.747748964624573 | 103230184 | PIGR | csab:103230184 |
| csab04973 | Carbohydrate digestion and absorption | 1/88 | 31/5900 | 0.373153286472273 | 0.747748964624573 | 103224900 | PIK3R3 | csab:103224900 |
| csab01522 | Endocrine resistance | 2/88 | 87/5900 | 0.373874482312286 | 0.747748964624573 | 103224900 103239621 | PIK3R3 NOTCH1 | csab:103224900 csab:103239621 |
| csab05222 | Small cell lung cancer | 2/88 | 87/5900 | 0.373874482312286 | 0.747748964624573 | 103218917 103224900 | TRAF1 PIK3R3 | csab:103218917 csab:103224900 |
| csab05167 | Kaposi sarcoma-associated herpesvirus infection | 3/88 | 148/5900 | 0.380351788389086 | 0.748736405825247 | 103230198 103235789 103224900 | IKBKE CXCL1 PIK3R3 | csab:103230198 csab:103235798 csab:103224900 |
| csab00051 | Fructose and mannose metabolism | 1/88 | 32/5900 | 0.382552248951475 | 0.748736405825247 | 103242625 | ALDOC | csab:103242625 |
| csab01200 | Carbon metabolism | 2/88 | 89/5900 | 0.38457824481024 | 0.748736405825247 | 103242625 103218486 | ALDOC ENO2 | csab:103242625 csab:103218486 |
| csab04130 | SNARE interactions in vesicular transport | 1/88 | 33/5900 | 0.391811860759974 | 0.756128152343809 | 103246629 | STX1A | csab:103246629 |
| csab04144 | Endocytosis | 4/88 | 214/5900 | 0.396704138443738 | 0.75891226484889 | 103246479 103233004 103223677 103239806 | FGFR3 EHD1 HSPA6 DNM1 | csab:103246479 csab:103233004 csab:103223677 csab:103239806 |
| csab04151 | PI3K-Akt signaling pathway | 5/88 | 280/5900 | 0.406554998247326 | 0.767518619984638 | 103237005 103245773 103246479 103224900 103223920 | IL7 SGK2 FGFR3 PIK3R3 IL6R | csab:103237005 csab:103245773 csab:103246479 csab:103224900 csab:103223920 |
| csab05165 | Human papillomavirus infection | 5/88 | 281/5900 | 0.409553197382924 | 0.767518619984638 | 103223483 103230198 103224207 103224900 103239621 | WNT7B IKBKE WNT2B PIK3R3 NOTCH1 | csab:103223483 csab:103230198 csab:103224207 csab:103224900 csab:103239621 |
| csab05170 | Human immunodeficiency virus 1 infection | 3/88 | 157/5900 | 0.416827089808476 | 0.767518619984638 | 103225587 103224900 103221664 | TNFRSF1B PIK3R3 LOC103221664 | csab:103225587 csab:103224900 csab:103221664 |
| csab00982 | Drug metabolism - cytochrome P450 | 1/88 | 36/5900 | 0.418774870974053 | 0.767518619984638 | 103230592 | FMO4 | csab:103230592 |
| csab04913 | Ovarian steroidogenesis | 1/88 | 36/5900 | 0.418774870974053 | 0.767518619984638 | 103235528 | PLA2G4C | csab:103235528 |
| csab03050 | Proteasome | 1/88 | 37/5900 | 0.427497212610186 | 0.767518619984638 | 103233253 | PSMB10 | csab:103233253 |
| csab00380 | Tryptophan metabolism | 1/88 | 38/5900 | 0.436090124991272 | 0.767518619984638 | 103232876 | LOC103232876 | csab:103232876 |
| csab04961 | Endocrine and other factor-regulated calcium reabsorption | 1/88 | 38/5900 | 0.436090124991272 | 0.767518619984638 | 103239806 | DNM1 | csab:103239806 |
| csab05204 | Chemical carcinogenesis - DNA adducts | 1/88 | 38/5900 | 0.436090124991272 | 0.767518619984638 | 103230154 | HSD11B1 | csab:103230154 |
| csab05219 | Bladder cancer | 1/88 | 38/5900 | 0.436090124991272 | 0.767518619984638 | 103246479 | FGFR3 | csab:103246479 |
| csab00140 | Steroid hormone biosynthesis | 1/88 | 39/5900 | 0.444555506942955 | 0.776208027995636 | 103230154 | HSD11B1 | csab:103230154 |
| csab04915 | Estrogen signaling pathway | 2/88 | 103/5900 | 0.457101874006638 | 0.790431049288526 | 103224900 103223677 | PIK3R3 HSPA6 | csab:103224900 csab:103223677 |
| csab04930 | Type II diabetes mellitus | 1/88 | 41/5900 | 0.461111137565473 | 0.790431049288526 | 103224900 | PIK3R3 | csab:103224900 |
| csab04217 | Necroptosis | 2/88 | 105/5900 | 0.467072892761402 | 0.790431049288526 | 103241278 103235528 | IL1B PLA2G4C | csab:103241278 csab:103235528 |
| csab04611 | Platelet activation | 2/88 | 105/5900 | 0.467072892761402 | 0.790431049288526 | 103235528 103224900 | PLA2G4C PIK3R3 | csab:103235528 csab:103224900 |
| csab05321 | Inflammatory bowel disease | 1/88 | 43/5900 | 0.477178751389335 | 0.801368895463005 | 103241278 | IL1B | csab:103241278 |
| csab04742 | Taste transduction | 1/88 | 44/5900 | 0.485034013448024 | 0.80341817291113 | 103229709 | SCNN1B | csab:103229709 |
| csab05022 | Pathways of neurodegeneration - multiple diseases | 6/88 | 373/5900 | 0.485702804532638 | 0.80341817291113 | 103241278 103223483 103225587 103224207 103246629 103227647 | IL1B WNT7B TNFRSF1B WNT2B STX1A UBA7 | csab:103241278 csab:103223483 csab:103225587 csab:103224207 csab:103246629 csab:103227647 |
| csab00280 | Valine, leucine and isoleucine degradation | 1/88 | 46/5900 | 0.500396145267222 | 0.809464352638153 | 103227954 | ABAT | csab:103227954 |
| csab00510 | N-Glycan biosynthesis | 1/88 | 46/5900 | 0.500396145267222 | 0.809464352638153 | 103242981 | MGAT5B | csab:103242981 |
| csab04730 | Long-term depression | 1/88 | 46/5900 | 0.500396145267222 | 0.809464352638153 | 103235528 | PLA2G4C | csab:103235528 |
| csab00561 | Glycerolipid metabolism | 1/88 | 47/5900 | 0.507906418450769 | 0.815590738711268 | 103224742 | PLPP3 | csab:103224742 |
| csab04923 | Regulation of lipolysis in adipocytes | 1/88 | 48/5900 | 0.515305057640301 | 0.815590738711268 | 103224900 | PIK3R3 | csab:103224900 |
| csab05031 | Amphetamine addiction | 1/88 | 48/5900 | 0.515305057640301 | 0.815590738711268 | 103246629 | STX1A | csab:103246629 |
| csab04613 | Neutrophil extracellular trap formation | 2/88 | 118/5900 | 0.529183857033287 | 0.831574632480879 | 103223257 103224900 | NCF4 PIK3R3 | csab:103223257 csab:103224900 |
| csab04330 | Notch signaling pathway | 1/88 | 51/5900 | 0.536847457964357 | 0.837634331575592 | 103239621 | NOTCH1 | csab:103239621 |
| csab04622 | RIG-I-like receptor signaling pathway | 1/88 | 52/5900 | 0.543815730096197 | 0.842531412825095 | 103230198 | IKBKE | csab:103230198 |
| csab04929 | GnRH secretion | 1/88 | 53/5900 | 0.550680336072862 | 0.845296427309052 | 103224900 | PIK3R3 | csab:103224900 |
| csab05213 | Endometrial cancer | 1/88 | 55/5900 | 0.564104626442885 | 0.845296427309052 | 103224900 | PIK3R3 | csab:103224900 |
| csab04934 | Cushing syndrome | 2/88 | 127/5900 | 0.569274269920209 | 0.845296427309052 | 103223483 103224207 | WNT7B WNT2B | csab:103223483 csab:103224207 |
| csab04918 | Thyroid hormone synthesis | 1/88 | 56/5900 | 0.570667294171375 | 0.845296427309052 | 103237469 | TG | csab:103237469 |
| csab04920 | Adipocytokine signaling pathway | 1/88 | 57/5900 | 0.577132263047645 | 0.845296427309052 | 103225587 | TNFRSF1B | csab:103225587 |
| csab05221 | Acute myeloid leukemia | 1/88 | 57/5900 | 0.577132263047645 | 0.845296427309052 | 103224900 | PIK3R3 | csab:103224900 |
| csab05152 | Tuberculosis | 2/88 | 129/5900 | 0.57784804320145 | 0.845296427309052 | 103241278 103231401 | IL1B ITGAX | csab:103241278 csab:103231401 |
| csab05218 | Melanoma | 1/88 | 58/5900 | 0.583500971048981 | 0.845296427309052 | 103224900 | PIK3R3 | csab:103224900 |
| csab04917 | Prolactin signaling pathway | 1/88 | 60/5900 | 0.595955252025395 | 0.845296427309052 | 103224900 | PIK3R3 | csab:103224900 |
| csab04218 | Cellular senescence | 2/88 | 134/5900 | 0.598743214982481 | 0.845296427309052 | 103230197 103224900 | RASSF5 PIK3R3 | csab:103230197 csab:103224900 |
| csab01524 | Platinum drug resistance | 1/88 | 61/5900 | 0.602043597542821 | 0.845296427309052 | 103224900 | PIK3R3 | csab:103224900 |
| csab04115 | p53 signaling pathway | 1/88 | 61/5900 | 0.602043597542821 | 0.845296427309052 | 103236967 | CD82 | csab:103236967 |
| csab04260 | Cardiac muscle contraction | 1/88 | 61/5900 | 0.602043597542821 | 0.845296427309052 | 103219220 | TPM2 | csab:103219220 |
| csab05214 | Glioma | 1/88 | 62/5900 | 0.608041227859011 | 0.845296427309052 | 103224900 | PIK3R3 | csab:103224900 |
| csab05322 | Systemic lupus erythematosus | 1/88 | 63/5900 | 0.613949479306151 | 0.845296427309052 | 103221732 | LOC103221732 | csab:103221732 |
| csab05161 | Hepatitis B | 2/88 | 138/5900 | 0.614903164608722 | 0.845296427309052 | 103230198 103224900 | IKBKE PIK3R3 | csab:103230198 csab:103224900 |
| csab04390 | Hippo signaling pathway | 2/88 | 142/5900 | 0.630568987646484 | 0.845296427309052 | 103223483 103224207 | WNT7B WNT2B | csab:103223483 csab:103224207 |
| csab03008 | Ribosome biogenesis in eukaryotes | 1/88 | 66/5900 | 0.631151033531186 | 0.845296427309052 | 103232396 | NXF3 | csab:103232396 |
| csab04146 | Peroxisome | 1/88 | 66/5900 | 0.631151033531186 | 0.845296427309052 | 103240818 | SOD2 | csab:103240818 |
| csab04740 | Olfactory transduction | 1/88 | 66/5900 | 0.631151033531186 | 0.845296427309052 | 103218440 | ANO2 | csab:103218440 |
| csab04520 | Adherens junction | 1/88 | 67/5900 | 0.636714747800857 | 0.845296427309052 | 103218491 | PTPN6 | csab:103218491 |
| csab04727 | GABAergic synapse | 1/88 | 67/5900 | 0.636714747800857 | 0.845296427309052 | 103227954 | ABAT | csab:103227954 |
| csab05207 | Chemical carcinogenesis - receptor activation | 2/88 | 146/5900 | 0.645742664557464 | 0.845296427309052 | 103230198 103224900 | IKBKE PIK3R3 | csab:103230198 csab:103224900 |
| csab03018 | RNA degradation | 1/88 | 69/5900 | 0.647594449336254 | 0.845296427309052 | 103218486 | ENO2 | csab:103218486 |
| csab04658 | Th1 and Th2 cell differentiation | 1/88 | 69/5900 | 0.647594449336254 | 0.845296427309052 | 103239621 | NOTCH1 | csab:103239621 |
| csab01232 | Nucleotide metabolism | 1/88 | 70/5900 | 0.652912866152993 | 0.845296427309052 | 103240330 | AMPD3 | csab:103240330 |
| csab05202 | Transcriptional misregulation in cancer | 2/88 | 148/5900 | 0.653145965348485 | 0.845296427309052 | 103215685 103218917 | PLAT TRAF1 | csab:103215685 csab:103218917 |
| csab05220 | Chronic myeloid leukemia | 1/88 | 71/5900 | 0.658151917229929 | 0.845296427309052 | 103224900 | PIK3R3 | csab:103224900 |
| csab05010 | Alzheimer disease | 4/88 | 302/5900 | 0.66699389798893 | 0.845296427309052 | 103241278 103223483 103224207 103224900 | IL1B WNT7B WNT2B PIK3R3 | csab:103241278 csab:103223483 csab:103224207 csab:103224900 |
| csab04912 | GnRH signaling pathway | 1/88 | 73/5900 | 0.668396588876839 | 0.845296427309052 | 103235528 | PLA2G4C | csab:103235528 |
| csab05212 | Pancreatic cancer | 1/88 | 73/5900 | 0.668396588876839 | 0.845296427309052 | 103224900 | PIK3R3 | csab:103224900 |
| csab05410 | Hypertrophic cardiomyopathy | 1/88 | 74/5900 | 0.673404500354244 | 0.845296427309052 | 103219220 | TPM2 | csab:103219220 |
| csab05203 | Viral carcinogenesis | 2/88 | 155/5900 | 0.678103779387178 | 0.845296427309052 | 103218917 103224900 | TRAF1 PIK3R3 | csab:103218917 csab:103224900 |
| csab04974 | Protein digestion and absorption | 1/88 | 75/5900 | 0.678337628395581 | 0.845296427309052 | 103242775 | COL20A1 | csab:103242775 |
| csab05414 | Dilated cardiomyopathy | 1/88 | 76/5900 | 0.683197077099647 | 0.845296427309052 | 103219220 | TPM2 | csab:103219220 |
| csab03015 | mRNA surveillance pathway | 1/88 | 79/5900 | 0.697344147856439 | 0.845296427309052 | 103232396 | NXF3 | csab:103232396 |
| csab04012 | ErbB signaling pathway | 1/88 | 79/5900 | 0.697344147856439 | 0.845296427309052 | 103224900 | PIK3R3 | csab:103224900 |
| csab04024 | cAMP signaling pathway | 2/88 | 161/5900 | 0.698333392503184 | 0.845296427309052 | 103223064 103224900 | ADORA2A PIK3R3 | csab:103223064 csab:103224900 |
| csab04914 | Progesterone-mediated oocyte maturation | 1/88 | 80/5900 | 0.701919601384807 | 0.845296427309052 | 103224900 | PIK3R3 | csab:103224900 |
| csab04512 | ECM-receptor interaction | 1/88 | 81/5900 | 0.706426658958371 | 0.845296427309052 | 103220724 | SDC1 | csab:103220724 |
| csab05210 | Colorectal cancer | 1/88 | 81/5900 | 0.706426658958371 | 0.845296427309052 | 103224900 | PIK3R3 | csab:103224900 |
| csab05016 | Huntington disease | 3/88 | 242/5900 | 0.706975193749389 | 0.845296427309052 | 103240818 103246306 103246629 | SOD2 TGM2 STX1A | csab:103240818 csab:103246306 csab:103246629 |
| csab04070 | Phosphatidylinositol signaling system | 1/88 | 83/5900 | 0.715239614815176 | 0.850555217618047 | 103224900 | PIK3R3 | csab:103224900 |
| csab04726 | Serotonergic synapse | 1/88 | 84/5900 | 0.719547490678381 | 0.851077677146472 | 103235528 | PLA2G4C | csab:103235528 |
| csab05415 | Diabetic cardiomyopathy | 2/88 | 169/5900 | 0.723680799982725 | 0.851389176450265 | 103223257 103224900 | NCF4 PIK3R3 | csab:103223257 csab:103224900 |
| csab04724 | Glutamatergic synapse | 1/88 | 87/5900 | 0.732088274228079 | 0.85294088832691 | 103235528 | PLA2G4C | csab:103235528 |
| csab03013 | Nucleocytoplasmic transport | 1/88 | 89/5900 | 0.740139117239143 | 0.85294088832691 | 103232396 | NXF3 | csab:103232396 |
| csab04725 | Cholinergic synapse | 1/88 | 89/5900 | 0.740139117239143 | 0.85294088832691 | 103224900 | PIK3R3 | csab:103224900 |
| csab05145 | Toxoplasmosis | 1/88 | 90/5900 | 0.744074370669354 | 0.85294088832691 | 103223677 | HSPA6 | csab:103223677 |
| csab04020 | Calcium signaling pathway | 2/88 | 176/5900 | 0.744384775267121 | 0.85294088832691 | 103246479 103223064 | FGFR3 ADORA2A | csab:103246479 csab:103223064 |
| csab05208 | Chemical carcinogenesis - reactive oxygen species | 2/88 | 188/5900 | 0.776828038990183 | 0.867480278040818 | 103240818 103224900 | SOD2 PIK3R3 | csab:103240818 csab:103224900 |
| csab04931 | Insulin resistance | 1/88 | 99/5900 | 0.77694197956947 | 0.867480278040818 | 103224900 | PIK3R3 | csab:103224900 |
| csab04145 | Phagosome | 1/88 | 100/5900 | 0.780325724750971 | 0.867480278040818 | 103223257 | NCF4 | csab:103223257 |
| csab04071 | Sphingolipid signaling pathway | 1/88 | 102/5900 | 0.786941699302186 | 0.867480278040818 | 103224900 | PIK3R3 | csab:103224900 |
| csab04722 | Neurotrophin signaling pathway | 1/88 | 102/5900 | 0.786941699302186 | 0.867480278040818 | 103224900 | PIK3R3 | csab:103224900 |
| csab04935 | Growth hormone synthesis, secretion and action | 1/88 | 102/5900 | 0.786941699302186 | 0.867480278040818 | 103224900 | PIK3R3 | csab:103224900 |
| csab03040 | Spliceosome | 1/88 | 104/5900 | 0.793360615898119 | 0.867480278040818 | 103223677 | HSPA6 | csab:103223677 |
| csab00230 | Purine metabolism | 1/88 | 106/5900 | 0.799588278470698 | 0.867480278040818 | 103240330 | AMPD3 | csab:103240330 |
| csab04514 | Cell adhesion molecules | 1/88 | 106/5900 | 0.799588278470698 | 0.867480278040818 | 103220724 | SDC1 | csab:103220724 |
| csab05012 | Parkinson disease | 2/88 | 198/5900 | 0.801094197005844 | 0.867480278040818 | 103223064 103227647 | ADORA2A UBA7 | csab:103223064 csab:103227647 |
| csab04152 | AMPK signaling pathway | 1/88 | 107/5900 | 0.802632156878461 | 0.867480278040818 | 103224900 | PIK3R3 | csab:103224900 |
| csab04910 | Insulin signaling pathway | 1/88 | 111/5900 | 0.814357274914559 | 0.867480278040818 | 103224900 | PIK3R3 | csab:103224900 |
| csab04926 | Relaxin signaling pathway | 1/88 | 111/5900 | 0.814357274914559 | 0.867480278040818 | 103224900 | PIK3R3 | csab:103224900 |
| csab04371 | Apelin signaling pathway | 1/88 | 113/5900 | 0.819958857773448 | 0.867480278040818 | 103215685 | PLAT | csab:103215685 |
| csab05017 | Spinocerebellar ataxia | 1/88 | 114/5900 | 0.822696652920491 | 0.867480278040818 | 103224900 | PIK3R3 | csab:103224900 |
| csab04261 | Adrenergic signaling in cardiomyocytes | 1/88 | 115/5900 | 0.825393281773411 | 0.867480278040818 | 103219220 | TPM2 | csab:103219220 |
| csab04080 | Neuroactive ligand-receptor interaction | 2/88 | 210/5900 | 0.827142991641116 | 0.867480278040818 | 103240389 103223064 | ADM ADORA2A | csab:103240389 csab:103223064 |
| csab05034 | Alcoholism | 1/88 | 116/5900 | 0.82804935631169 | 0.867480278040818 | 103223064 | ADORA2A | csab:103223064 |
| csab01240 | Biosynthesis of cofactors | 1/88 | 121/5900 | 0.840742211285226 | 0.874984951510617 | 103225584 | DHRS3 | csab:103225584 |
| csab04921 | Oxytocin signaling pathway | 1/88 | 122/5900 | 0.843167316910231 | 0.874984951510617 | 103235528 | PLA2G4C | csab:103235528 |
| csab04140 | Autophagy - animal | 1/88 | 126/5900 | 0.852507984492901 | 0.880524678818958 | 103224900 | PIK3R3 | csab:103224900 |
| csab04120 | Ubiquitin mediated proteolysis | 1/88 | 130/5900 | 0.861298286509064 | 0.885446836598103 | 103227647 | UBA7 | csab:103227647 |
| csab04141 | Protein processing in endoplasmic reticulum | 1/88 | 147/5900 | 0.893228569674666 | 0.914001327108961 | 103223677 | HSPA6 | csab:103223677 |
| csab04360 | Axon guidance | 1/88 | 161/5900 | 0.913974789829869 | 0.930900248900792 | 103224900 | PIK3R3 | csab:103224900 |
| csab05014 | Amyotrophic lateral sclerosis | 2/88 | 284/5900 | 0.930471255083671 | 0.943092245975754 | 103225587 103232396 | TNFRSF1B NXF3 | csab:103225587 csab:103232396 |
| csab04510 | Focal adhesion | 1/88 | 182/5900 | 0.93784895873177 | 0.943092245975754 | 103224900 | PIK3R3 | csab:103224900 |
| csab05166 | Human T-cell leukemia virus 1 infection | 1/88 | 183/5900 | 0.938805463039501 | 0.943092245975754 | 103224900 | PIK3R3 | csab:103224900 |
| csab05132 | Salmonella infection | 1/88 | 199/5900 | 0.952271390193144 | 0.952271390193144 | 103241278 | IL1B | csab:103241278 |
